# Transcriptome profiling of a *Sinorhizobium meliloti fadD *mutant reveals the role of rhizobactin 1021 biosynthesis and regulation genes in the control of swarming

**DOI:** 10.1186/1471-2164-11-157

**Published:** 2010-03-08

**Authors:** Joaquina Nogales, Ana Domínguez-Ferreras, Carol V Amaya-Gómez, Pieter van Dillewijn, Virginia Cuéllar, Juan Sanjuán, José Olivares, María J Soto

**Affiliations:** 1 Departamento de Microbiología del Suelo y Sistemas Simbióticos, Estación Experimental del Zaidín, CSIC, Profesor Albareda, 1, 18008 Granada, Spain

## Abstract

**Background:**

Swarming is a multicellular phenomenom characterized by the coordinated and rapid movement of bacteria across semisolid surfaces. In *Sinorhizobium meliloti *this type of motility has been described in a *fadD *mutant. To gain insights into the mechanisms underlying the process of swarming in rhizobia, we compared the transcriptome of a *S. meliloti fadD *mutant grown under swarming inducing conditions (semisolid medium) to those of cells grown under non-swarming conditions (broth and solid medium).

**Results:**

More than a thousand genes were identified as differentially expressed in response to growth on agar surfaces including genes for several metabolic activities, iron uptake, chemotaxis, motility and stress-related genes. Under swarming-specific conditions, the most remarkable response was the up-regulation of iron-related genes. We demonstrate that the pSymA plasmid and specifically genes required for the biosynthesis of the siderophore rhizobactin 1021 are essential for swarming of a *S. meliloti *wild-type strain but not in a *fadD *mutant. Moreover, high iron conditions inhibit swarming of the wild-type strain but not in mutants lacking either the iron limitation response regulator RirA or FadD.

**Conclusions:**

The present work represents the first transcriptomic study of rhizobium growth on surfaces including swarming inducing conditions. The results have revealed major changes in the physiology of *S. meliloti *cells grown on a surface relative to liquid cultures. Moreover, analysis of genes responding to swarming inducing conditions led to the demonstration that iron and genes involved in rhizobactin 1021 synthesis play a role in the surface motility shown by *S. meliloti *which can be circumvented in a *fadD *mutant. This work opens a way to the identification of new traits and regulatory networks involved in swarming by rhizobia.

## Background

Swarming is a type of bacterial motility generally dependent on flagella and is characterized by a rapid and co-ordinated population migration across solid surfaces. In contrast to other modes of bacterial surface translocation, swarming involves a complex process of differentiation in which cells usually become hyperflagellated and elongated [1]. Signals and signalling pathways controlling swarm cell differentiation are largely unknown. Extracellular chemical signals such as N-acyl-homoserine lactones (AHL), peptides and amino acids, fatty acids, polyamines, etc, as well as physiological parameters, surface contact and wetness provide stimuli to trigger swarm cell differentiation (reviewed in [1-4]). It is generally believed that the different environmental, cell-to-cell, and intracellular signals may be sensed and transduced by two-component regulatory systems and cytosolic regulators, leading to a complex regulatory network.

Classical genetic studies performed in different bacteria have allowed the identification of several genes essential for swarming. Interestingly, recent genome-scale approaches performed in model bacteria such as *Salmonella typhimurium*, *Escherichia coli *and *Pseudomonas aeruginosa*, indicate that swarmer differentiation represents much more than a motility phenotype as substantial alterations in metabolic pathways and gene expression have been observed [5-9]. In *E. coli*, up to one-fifth of the genes on the genome seem to be involved in swarming [7]. Besides flagellar functions, a large number of genes involved in several metabolic activities, iron acquisition, regulatory proteins, chaperones, and biosynthesis of cell surface components have been demonstrated to be important for this multicellular migration [7,8].

In several pathogenic bacteria, swarming is associated with virulence [1,2]. This could be partially due to the fact that the expression of some virulence determinants seems to be coregulated with swarmer differentiation. Urease, metalloprotease and haemolysin are up-regulated during swarming in the uropathogenic *Proteus mirabilis *[3], whereas phospholipase is induced in the opportunistic pathogen *Serratia liquefaciens *[10]. Global gene expression analysis performed on swarmer cells has revealed the up-regulation of a large number of virulence-related genes in *S. typhimurium *and *P. aeruginosa *such as genes encoding components of a type III secretion system, its effectors, extracellular proteases, and proteins involved in iron transport [6,9]. An interesting aspect related to virulence is the fact that swarmer cells, like biofilm communities, display increased resistance to several antimicrobials when compared to planktonic cells [9,11].

Although swarming has been extensively studied in pathogenic bacteria, this type of surface motility has also been described in beneficial bacteria such as rhizobia. These soil bacteria are known for their ability to establish a mutualistic symbiosis with legume plants. A remarkable feature of this interaction is the formation of a new organ, the root nodule, within which endosymbiotic differentiated bacteria fix atmospheric nitrogen to generate nitrogen sources usable by the plant, thus conferring a nutritional advantage to the host. The formation of a nitrogen-fixing nodule is a complex process requiring the coordination of bacterial infection with a root developmental program (for a review see [12,13]). Accumulating evidence suggests that in order to colonize, invade and establish a chronic infection within the host, rhizobia use similar strategies as pathogenic bacteria (reviewed in [14,15]).

The first report of swarming by rhizobia was described for a *fadD *mutant of the alfalfa symbiont *Sinorhizobium meliloti *[16]. In this bacterium, the lack of the *fadD *gene (encoding a long-chain fatty acyl-coenzyme A ligase), results in multicellular swarming behaviour but also defects in nodulation, thereby suggesting that fatty acid-related compounds may act as signals controlling motility and symbiosis. More recently, it has been reported that a wild type strain of *Rhizobium etli*, the bacterial symbiotic partner of common bean plants, can swarm [17]. The finding that mutants in the *cinIR *quorum sensing system of this bacterium were no longer able to move over semisolid surfaces, led to the discovery that AHL carrying a long-chain fatty acid moiety have a dual role in swarming in this rhizobium: as quorum sensing signals and as biosurfactants which promote surface translocation [18]. The characterization of several *R. etli *mutants defective in swarming has allowed the identification of additional genetic determinants which seem to play a role in this multicellular behaviour, including genes involved in polysaccharide synthesis or export, motility and amino acid and polyamines metabolism [19]. Interestingly, half of the mutants with an altered swarming pattern showed deficiencies in either nodulation or nitrogen fixation. The biological role of swarming in rhizobia remains to be elucidated. However, the fact that some mutations which alter swarming behaviour in *S. meliloti *and *R. etli *result in an impairment in the establishment of the symbiosis, suggests either that components essential for this multicellular motility and/or factors which are co-regulated during swarmer cell differentiation may play a role in the interaction with the host plant.

To gain insights into the adaptation process involved in multicellular swarming motility in rhizobia, global gene expression profiles of *S. meliloti fadD *cells under swarming inducing conditions were determined and compared with the profiles obtained during growth in liquid media as well as on non-swarming hard agar.

## Results and Discussion

### Construction and characterization of a *S. meliloti *Rm1021 *fadD *mutant

**Figure 1:**
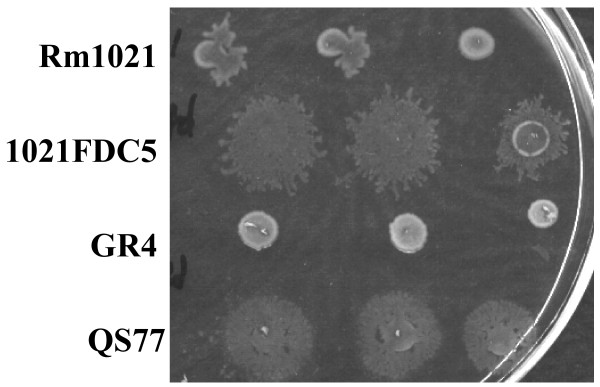
**Swarming behaviour of *S. meliloti *strains**. The swarming motility phenotype of *S. meliloti *wild-type strains Rm1021 and GR4 and their corresponding *fadD*-derivative mutants 1021FDC5 and QS77 was analyzed by inoculating aliquots (2 μl) of each strain prepared as described in Materials and Methods onto semisolid MM plates containing 0.6% purified agar. Replicates corresponding to the same strain were placed in a row. The photograph was taken 48 hours after inoculation and is a representative of at least three independent experiments.

In *S. meliloti*, swarming motility has been reported for a *fadD *mutant (QS77) of the GR4 strain. Under the same swarming inducing conditions, the wild type strain GR4 has never shown this surface motility [16,20]. In order to identify *S. meliloti *genes whose expression is altered under swarming inducing conditions, we performed a transcriptomic analysis of a *fadD *mutant using the Sm6kOligo microarrays [21]. Since these arrays are based on the genome of *S. meliloti *strain Rm1021 [22], we constructed a *fadD *mutant in this genetic background by site-directed mutagenesis as described in Methods. The mutant obtained was named 1021FDC5. In contrast to the wild type strains GR4 and Rm1021, 1021FDC5 like QS77 could not grow on minimal medium (MM) plates containing oleate as sole carbon source (data not shown), a phenotype that was restored after introduction of the pBBRD4 construct harbouring a wild type *fadD *gene. Furthermore, as reported for QS77, 1021FDC5 showed conditional swarming motility on semisolid MM plates (Fig. 1). It is worth mentioning that whereas GR4 has never shown surface motility under our swarming inducing conditions, in approximately 70% of the experiments performed, Rm1021 cells spread over the surface of the plate resembling the movement displayed by the *fadD *mutants (Fig. 1). A similar behaviour was observed for the closely related strain Rm2011 (see below). This suggests that the control of swarming may be different in GR4 and Rm1021/Rm2011, although in all three *S. meliloti *strains a mutation in *fadD *promotes multicellular surface motility (Fig. 1; see below). This result was particularly intriguing as it has been published that in *S. meliloti*, ExpR is required for swarming but not for swimming [23,24], and it is well known that Rm1021 and Rm2011 are *expR*-deficient strains [25]. We have tried to reproduce swarming in different *S. meliloti *strains under the conditions described by Bahlawane *et al*. [24] without success. In any case, we show here that *expR*-defective strains (Rm1021, Rm2011 and their *fadD*-derivative mutants) can swarm on semisolid MM which suggests that the role of ExpR in swarming needs to be re-evaluated.

### Transcriptome profiling of *S. meliloti *1021FDC5 in broth and on agar surfaces

**Figure 2:**
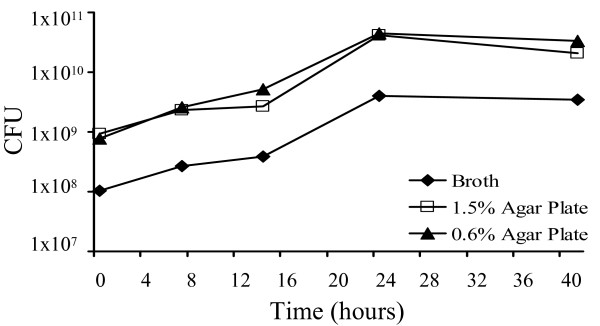
**Growth curves of *S. meliloti *1021FDC5 in broth and agar surfaces**. Bacterial growth curves were determined in liquid, semisolid (0.6% purified agar) and solid (1.3% purified agar) MM. CFU refers to colony forming units/ml of broth or per plate. Data are representative of at least two replicate experiments.

**Figure 3:**
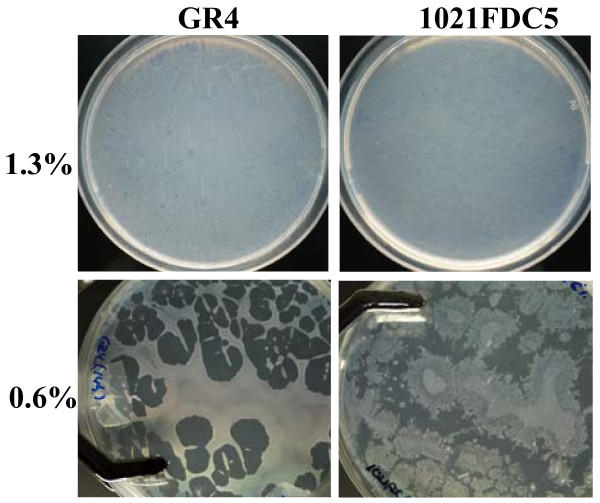
**Macroscopic appearance of 1021FDC5 cells growing on solid and semisolid MM plates**. 100 μl of a suspension containing approximately 10^9 ^cells of either *S. meliloti *1021FDC5 (*fadD *mutant) or GR4 (wild type strain which does not show swarming) were evenly spread over the surface of solid (1.3% agar) and semisolid (0.6% agar) MM plates. The photographs were taken 14 hours after incubation at 30°C.

In order to identify genes whose expression is altered during swarming in *S. meliloti*, the transcriptome of 1021FDC5 growing on swarming inducing media (semisolid MM containing 0.6% agar) was compared with that of cells growing under non-swarming conditions (solid MM containing 1.3% hard agar and MM broth). We also compared the transcriptomes of 1021FDC5 after growth in broth and on solid MM to identify genes which are not specific for swarming but responsive to growth on surfaces. This analysis required, as a first step, the determination of bacterial growth curves in liquid, semisolid and solid MM to ensure that the respective transcriptomes were obtained in the same growth phase. Fig. 2 shows that the growth profiles were very similar for all three conditions tested, with cells entering stationary phase at 24 h. We studied the different expression profiles at early exponential phase (7 hours) and mid exponential phase (14 hours). In our standard swarming assays, 7 hours is the minimum time required to macroscopically observe surface motility whereas after 14 hours, swarming diminishes as recognized by slower cell migration and increased mucoidy. The macroscopic appearance of 1021FDC5 cells growing on solid and semisolid MM is shown in Fig. 3. After 14 h on solid MM (1.3% agar), 1021FDC5 grows as a homogenous lawn on the plate, indistinguishable from the non-swarming strain GR4. On the other hand, on semisolid MM (0.6%), growth of both GR4 and 1021FDC5 is not homogenous on the surface of the plate with visible uncolonized areas. However, whereas the borders of colonized areas by GR4 are smooth, in the case of 1021FDC5 these borders show a dendritic morphology indicating that these cells were actively swarming. Therefore, we conclude that this experimental setup is adequate for a transcriptomic study of swarming.

**Figure 4:**
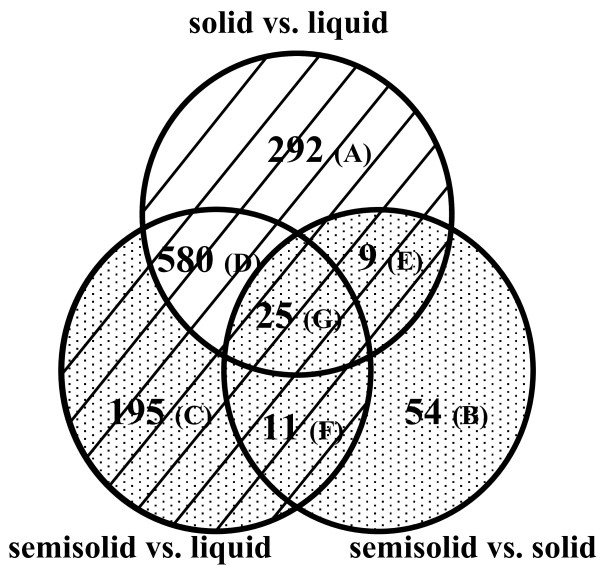
**Venn diagram of *S. meliloti *differentially regulated genes identified in microarray experiments**. The diagram represents the number of differentially expressed genes obtained in six different microarray experiments (based on additional file 1). The transcriptomes of *S. meliloti *1021FDC5 cells obtained after growth in liquid, solid or semisolid MM were compared. For each of the three combinations, changes in the mRNA levels were monitored after 7 and 14 hours of growth. The different subsets have been assigned with letters (A-G). Surface responsive genes are indicated with stripes and swarming responsive genes with dots.

An internal control experiment in which Cy3- and Cy5- labelled cDNAs were synthesized from total RNA extracted from liquid cultures of 1021FDC5, allowed us to consider as differentially expressed only genes showing an M value of ≥ 1 or ≤ -1. A total of 1166 genes (19% of the annotated genes in the *S. meliloti *Rm1021 genome) appeared as differentially expressed in any of the six conditions studied (see additional file 1 and Table 1). More than 35% of the 1166 genes formed part of presumed operons where two or more genes appeared as differentially expressed under our experimental conditions. To facilitate the analysis, genes showing up- or down-regulation in any of the six different comparisons were plotted in a Venn diagram (Fig. 4). Most of the genes identified in our study (1112) showed differential expression in response to growth on a surface (i.e. differentially expressed in cells grown on solid or semisolid media vs. broth; striped area in Fig. 4) and only 54 genes appeared exclusively in the comparison of the transcriptome of cells grown on semisolid MM with that of cells grown on solid MM. Within the group of surface-responsive genes, more than 50% of the genes (580 + 25) showed differential expression regardless of the concentration of agar used, whereas a smaller number of genes showed differential expression after growth specifically on hard agar or semisolid medium, (292 and 195, respectively). On the other hand, a total of 294 genes were found to be differentially expressed specifically under swarming inducing conditions (dotted area in Fig. 4 comprising genes which appeared differentially expressed in the comparison of cells grown on semisolid vs. solid media (99 genes), plus 195 genes which exclusively appeared differentially expressed in cells grown on semisolid vs. liquid media). It is noteworthy that 45 out of the 294 genes differentially expressed under swarming inducing conditions were also found differentially expressed in response to surface growth (subsets E, F, and G of 9, 11, and 25 genes, respectively; Fig. 4). This might suggest that a significant portion of swarming-responsive genes are regulated in response to contact with a surface, a known signal for swarming in other bacteria [1,4].

**Table 1:** Number of genes differentially expressed in *S. meliloti *1021FDC5 in response to different growth conditions

	Up-regulated		Down-regulated	
	
Comparison	7 hours	14 hours	7 hours	14 hours
Semisolid vs liquid	35	580	8	217
Semisolid vs solid	38	18	39	9
Solid vs liquid	7	542	10	354

### Surface responsive genes

The comparison of the transcriptome of cells grown in liquid MM with that of cells grown on solid or semisolid MM sampled at two different time points, led to the identification of 1112 differentially expressed genes (Table 1 and striped area in Fig. 4): 705 genes were up-regulated in response to surface growth, 384 were down-regulated, and 23 genes showed variable responses (up- or down-regulated depending on the time point or agar concentration). Most of the surface responsive genes identified in our study (96%) showed a late response, appearing as differentially expressed after 14 hours growth (Table 1).

Many of the down-regulated genes (31%) encoded proteins of unknown or hypothetical function, which hindered drawing conclusions from down-regulated processes. Most noteworthy of the remaining down-regulated genes is that several are involved in nitrogen metabolism and exopolysaccharide production. Among the former are the regulatory genes *glnK *and *ntrBC*, glutamine synthetase genes (*glnII*, *glnT*, SMc01594 and SMc02352), putative glutamate synthase genes (*glxBCD *and *gltD*), the *nirB *nitrite reductase gene, and genes coding for transporters for ammonium (*amtB*), nitrate (*nrtABC *and SMb21114), and amino acids (*aap *genes). The lower expression observed for most of these genes could be explained by the down-regulation of the *ntrC *gene coding for the key transcriptional activator of nitrogen catabolic operons [26]. Likewise, the expression of some *nif *(*nifA, nifB, nifX*) and *fix *(*fixB, fixI*_2_, *fixQP*_3_) genes was diminished in cells grown on solid and semisolid media compared to liquid culture. This could also be a consequence of the lower abundance of the NtrC activator and/or of the higher oxygen concentration in agar-solidified media. The other conspicuous group of down-regulated genes in response to growth in agar surfaces included several *exo *genes involved in exopolysaccharide (EPS) production (*exoA*, *exoM*, *exoN*, *exoP*, *exoN2*).

In contrast with the down-regulated genes, the majority (85%) of the genes up-regulated in response to surface growth have known or putative functions. Below is a description of the most relevant ones:

#### 1) Carbon and energy metabolism

The induction of genes involved in the uptake (*smoEFGK*) and metabolism (*smoS*, *mtlK*, *xylA*) of mannitol as well as those involved in glutamate degradation (*glmS*, *gsh1*, *carA*, *gabT*, *nodM*), the carbon and nitrogen sources provided in our experiments, indicated a higher metabolic rate in response to surface growth. This is in agreement with the up-regulation of genes of the tricarboxylic acid cycle (*lpdA2*, *acnA*, *icd*, *sdhBCD*, *mdh*, *sucABCD*, *pckA*), the Calvin cycle (SMb20194, *ppe*, *cbbXSLAT*), glycolysis (*cbbA2*, *gap*, *glk*, *pgk*, *eno, pdhAa*, *pgi*), and of the different complexes in the respiratory chain and associated functions: *nuoA1B1C1D1G1IJK1LMN*, *cyoBC*, *fixN1Q1*, *ndh*, *ctaBCDE*, *rrpP*, *ppa, ppk, atpABDEFF2GHI *and SMc00410. The higher metabolic rate could also be the cause of the observed induction of phosphate transport systems (*phoTEDC*, *phoU*, *pstABC *and SMc02146).

#### 2) Protein metabolism

As many as 54 genes coding for ribosomal proteins were found to be induced during surface growth. We also observed up-regulation of different genes involved in the ribosome assembly and maturation (*rbfA *and *rhlE*), genes involved in the processing of mRNA, rRNA and tRNA (*rne*, *rnc*, *rnr*, *rnpA *and *pnp*), and different genes related to the translation process (*infB*, *tufA, tufB, fusA1, tsf*, *pth *and *prfB*). Due to the general induction of protein synthesis it was not a surprise to find induction of other related processes such as tRNA and amino acid biosynthesis (16 tRNA synthetases and 46 genes involved in the synthesis of different amino acids showed increased expression during surface growth).

#### 3) Macromolecule synthesis

In agreement with the above mentioned increase in protein synthesis, the induction of several functions related to the transcription process was also observed, including the induction of RNA polymerase genes (*rpoA*, *rpoB*, *rpoC *and *rpoZ*), several sigma factors (*rpoH1*, *rpoE4 *and *sigA*) and the transcription terminator factor (*rho*). On the other hand, we also detected induction of genes involved in DNA synthesis (*dnaN*, *dnaX*, *topA*, and *gyrA*) and related functions (*purA*, *purM*, *purQ*, *guaA*, *guaB*, *pyrB*, SMc01361, *pyrC-pyrE-frk*, *pyrF*, *cyaC*, SMa2357, *ndk, prsA*, SMc02218,*typA*).

Our microarray data suggest that during growth on agar surfaces, *S. meliloti *cells stimulate fatty acid biosynthesis over degradation. Thus, genes involved in the initiation (*accA*, *accBC*, *accD*) and elongation (*fabABI2*, *fabF*, *fabG*, *plsX-fabH*, *fabI1*, *fabZ*, SMc04273) of fatty acids and the acyl carrier protein AcpP were up-regulated during growth on agar media compared to broth, whereas the *fadB *and SMc02229 genes, putatively involved in degradation of fatty acids were down-regulated.

As previously mentioned, we observed repression of several *exo *genes suggesting that in response to growth on agar surfaces, *S. meliloti *produces less succinoglycan. On the contrary, several genes with a role in the synthesis of different surface polysaccharides were found to be up-regulated. This was the case for the *kdsA*, *kdsB *and *kdtA *genes, involved in the synthesis and transfer of Kdo (3-deoxy-D-manno-2-octulosonic acid), a component present in capsular polysaccharides (KPS) and lipopolysaccharides (LPS); the *rkpA *gene involved in the biosynthesis of a specific lipid carrier required for KPS synthesis; and the *acpXL *and *lpxD *genes involved in the biosynthesis of the lipid A of LPS [27]. Genes involved in the transport and modification of cyclic β-glucans such as *ndvA *and *opgC *as well as genes involved in the synthesis of peptidoglycan (*murA*) and lipoproteins (*lgt*) were also up-regulated under surface growth conditions.

#### 4) Motility and chemotaxis

No less than thirty seven genes of the flagellar regulon were up-regulated during growth on a surface, whereas only two chemotaxis genes (*cheW3 *and *mcpT*) showed lower expression under these conditions compared to growth in liquid medium. Up-regulated genes included those for chemotaxis (*cheABR *and *mcpEUX*), the flagellar structure (*flaCD, fliEFLGM*, *fliK*, *flgABCDEFGHIKL*), the flagellar motor (*motABC*), the chaperone-encoding gene *motE*, related genes of yet unknown function (SMc03013, SMc03017, SMc03023, and SMc03045), as well as genes coding for regulatory proteins (*flaF*, *flbT*, *visN *and *rem*) [28,29]. Motility genes were generally more induced than chemotaxis genes in response to growth on a surface. Five genes belonging to the four different classes of the *S. meliloti *flagellar regulon were chosen to validate our microarray data (see below).

#### 5) Iron uptake and metabolism

19 genes up-regulated in response to growth on surfaces belong to this functional category, including genes involved in the synthesis (*rhbBCDEF *and SMa2339) and transport (*rhtA, rhtX*) of the siderophore rhizobactin 1021 [30-32]; several genes coding for proteins involved in the uptake of haem and hydroxamate siderophores (*hmuPS*, *hmuT, shmR, fhuA1, fhuA2, fhuP*) [33-35]; the *exbB-exbD *genes putatively coding for the inner membrane components of the TonB energy transduction complex required for Fe^3+^-siderophore acquisition systems [36]; the *fhuF *gene coding for ferrioxamine B reductase [35]; and the putative iron regulator *irr*. Induction of these genes may be related to increased difficulty for iron acquisition during growth on a solid surface due to a slower diffusion of nutrients than in broth.

#### 6) Stress-related genes

Up-regulation of genes related to oxidative stress was detected in response to surface growth including *sodB*, *katA*, peroxidases (SMb20964 and *cpo*), and glutathione transferases (*gst4 *and *gst8*). Noticeable was also the induction of genes related to thermal stress such as those coding for cold shock proteins (*cspA1*, *cspA4*, *cspA2 *and *cspA6*) and heat shock proteins (*grpE*, *hslU*, *hslV*, *hslO*, *ibpA*, SMb21295 and SMc01106). The up-regulation of genes involved in DNA repair processes (*radA*, *recF*, *recN *and *ligA*) could be linked to the induction of genes involved in DNA synthesis (see above), whereas the induction of chaperone genes (*groESL1, groESL2, tig, ibpA*, *lon*) could be the consequence of the observed increase in protein synthesis and/or the existence of stress conditions during surface growth. Also noteworthy was the induction of several genes involved in resistance to different toxic compounds. This was the case for *mrcA1*, a gene coding for a probable penicillin-binding 1A transmembrane protein, the *fsr *gene which encodes a putative fosmidomycin resistance transmembrane protein, the *uppP *gene coding for a putative undecaprenyl-diphosphatase which could confer resistance to bacitracin, putative components of a multidrug efflux system (SMc02867 and SMc02868), and the *aqpS*-*arsC *genes involved in arsenic detoxification.

All together these data suggest the existence of striking differences in the physiology of *S. meliloti *growing in broth compared with agar surfaces and more specifically that cells growing on agar surfaces have a higher metabolic rate than those grown in broth. Similar results were obtained in a transcriptomic study performed in *Salmonella *[6]. As suggested in the work by Wang *et al*. [6], this could be explained if agar surfaces represent a more aerobic environment than liquid cultures. This could also explain the down-regulation we have observed for several low oxygen responsive genes (*nif *and *fix*) during growth on agar-solidified media when compared to broth. On the other hand, the up-regulation of several genes related to oxidative stress, chaperone functions, or genes involved in resistance to different toxic compounds, could indicate that cells growing on solid agar surfaces are subject to stress. However, the observed induction of chemotaxis and motility genes together with the down-regulation of several *exo *genes under surface growth contrast with the response of *S. meliloti *to several environmental stresses (osmotic stress, phosphate and iron starvation, or acidic pH), in which motility genes are down-regulated while at the same time *exo *genes are up-regulated [21,37,38]. The identification of several regulatory genes in *S. meliloti *which simultaneously affect EPS production and cellular motility, indicates that regulation of these two rhizobial traits are coupled [24,39-41]. In addition to environmental stresses, the results obtained in this work suggest that contact with a surface might be another signal recognized by *S. meliloti *to co-ordinate the regulation of EPS production and motility.

### Regulation of genes in response to swarming-specific conditions

In contrast to surface growth, our microarray data revealed that the response of *S. meliloti *to swarming-specific conditions is characterized by the differential expression of a smaller number of genes (294) (dotted area in Fig. 4; additional file 2): 99 of these were identified in the comparison semisolid vs. solid, 36 of which also appeared in the comparison semisolid vs. broth, plus 195 genes which exclusively appeared in semisolid vs. broth. This result is comparable to that found in a similar transcriptomic study performed in *Salmonella *in which a small number of genes (97) were found to show swarming-specific regulation, in contrast with more than a thousand genes found to respond to surface growth [6]. In our study, most of the genes (73%) responding to swarming-specific conditions identified in the comparison semisolid vs. solid showed an early response (7 hours after incubation) (Table 1). On the contrary, the majority of genes (89%) identified in the semisolid vs. broth comparison, appeared after 14 hours of growth.

207 genes out of the 294 genes were up-regulated under swarming-inducing conditions, only 78 were found to be down-regulated and the remaining 9 showed variable responses. No informative conclusions could be reached from down-regulated functions as, firstly, approximately one fourth of the genes code for hypothetical proteins of unknown function and secondly the remaining down-regulated genes belong to diverse functional categories. Similarly, many of the up-regulated genes have unknown functions or display partial or global homology to genes deposited in databases (66 genes). This suggests that bacterial components with a putative role in swarming in *S. meliloti *have yet to be thoroughly studied. However, a subset (25 genes) of the up-regulated genes induced under swarming inducing conditions could be assigned to iron uptake and metabolism, including the transcriptional regulator of the iron limitation response *rirA *[42,43], and the putative iron response regulator *irr*. It is also interesting that swarming conditions induced in *S. meliloti *1021FDC5 a slight up-regulation of genes involved in the resistance to toxic compounds (*mrcA2, uppP, aqpS-arsC*). Increased resistance to antibiotics and to other antimicrobials has been observed in swarmer cells of different bacteria [9,11]. Whether this is also the case for *S. meliloti *swarmer cells will be the subject of future studies.

To gain further insight into some of the genes responding to swarming specific conditions, we focused on the subset of 36 genes (from now on S36) which were identified as differentially expressed in both semisolid vs. solid and semisolid vs. broth (subsets F and G of 11 and 25 genes, respectively in Fig. 4) (Table 2). Five genes of S36 were chosen to validate our microarray data (see below). The majority of the genes within S36 (27 genes) were up-regulated under swarming inducing conditions compared to growth in either broth or on hard agar, whereas only one gene (SMb21284) was found to be down-regulated. 36% of the genes belonging to S36 were located on megaplasmid pSymA, a percentage which is significantly higher than the 21% expected for an even distribution among *S. meliloti *replicons, suggesting a putative role of this megaplasmid in *S. meliloti *swarming. Interestingly, up to 17 genes present in S36 are related with iron uptake and metabolism. They include SMb21431 and SMb21432 which code for putative components of iron uptake ABC transporters, and 15 out of the 19 iron-related genes identified as up-regulated in response to surface growth (only *rhbD, rhtX, fhuA1, fhuA2 *and *irr *were not present in S36). 13 out of the 15 surface-responsive genes related to iron uptake and metabolism were also significantly induced on solid MM versus broth after 14 hours of growth (see additional file 1). Although the induction of these genes could be explained by the increased difficulty for iron acquisition during growth on agar containing media, it is surprising that up to 8 of these iron-related genes show higher expression in 0.6% swarm agar than in the harder 1.3% agar. This suggests that lower diffusion of iron is not the only factor controlling the expression of genes involved in iron uptake and metabolism, and furthermore that a specific connection may exist between swarming and iron-related genes. In *S. typhimurium *and *P. aeruginosa *induction of genes related to iron uptake and metabolism has also been detected in the transcriptomic analysis of swarmer cells [6,9]. Moreover, mutants of *E. coli *and *P. putida *affected in different systems of iron acquisition show defects in swarming [7,44]. These and our results suggest that different bacteria have acquired similar adaptation processes for swarming with iron acquisition systems playing an important role.

**Table 2:** Subset S36^a ^of *S. meliloti *1021FDC5 genes differentially expressed under swarming-specific conditions

Gene	Descriptions	M value^b^			
		
		SS/L 7 h	SS/L 14 h	SS/S 7 h	SS/S 14 h
SMa0520	Transcriptional regulator, RpiR family	**1,45**	**1,90**	**1,73**	**1,55**
SMa0564	Putative dehydrogenase	-0,45	**-1,12**	-0,83	**2,78**
SMa1052	Conserved hypothetical protein	**1,01**	**1,24**	0,51	**1,56**
SMa1077 (*nex18*)^c^	Nex18 Symbiotically induced conserved protein	**1,16**	0,81	0,44	**2,76**
SMa1078	Conserved hypothetical protein	**1,89**	**1,74**	0,28	**1,93**
SMa1079 (*tspO*)	TspO Tryptophan rich sensory protein	**1,36**	0,31	0,59	**1,91**
SMa1100	Conserved hypothetical protein	**1,31**	**1,61**	0,52	**1,57**
SMa2339	Siderophore biosynthesis protein	0,80	**1,33**	**1,55**	-0,17
SMa2402 (*rhbB*)^c^	L-2,4-diaminobutyrate decarboxylase	**1,84**	0,75	**2,58**	-0,19
SMa2404 (*rhbC*)	RhbC rhizobactin biosynthesis protein	1,49	**1,19**	**2,65**	0,00
SMa2408 (*rhbE*)	RhbE rhizobactin biosynthesis protein	**2,38**	**2,23**	**3,83**	0,03
SMa2410 (*rhbF*)	RhbF rhizobactin biosynthesis protein	**2,36**	**1,34**	**3,76**	-0,11
SMa2414 (*rhtA*)^c^	RhtA rhizobactin transporter	**1,43**	**1,64**	**2,68**	0,05
SMb20005	Putative glutathione S-transferase	**2,34**	-0,08	0,31	**-1,38**
SMb20604	ABC transporter, permease	0,18	**-4,84**	0,20	**1,14**
SMb20605	ABC transporter, periplasmic solute-binding protein	0,01	**-5,55**	0,09	**1,34**
SMb21284	Putative polysaccharide deacetylase	-0,21	**-1,55**	-0,15	**-1,15**
SMb21431	Hypothetical protein, possibly C terminus of iron ABC transporter periplasmatic solute-binding protein	-0,34	**1,75**	**1,44**	-0,14
SMb21432	Putative iron uptake ABC transporter periplasmic solute-binding protein precursor	**-1,07**	**1,63**	**2,15**	-0,40
SMb21676	Hypothetical protein	0,17	**1,92**	-0,58	**1,96**
SMc00402	Hypothetical signal peptide protein	-0,03	**1,91**	**1,42**	-0,23
SMc00592	Hypothetical, transmembrane protein	-0,44	**1,47**	**1,30**	-0,23
SMc01242	Conserved hypothetical signal peptide protein	0,29	**-1,35**	0,13	**1,04**
SMc01417	Hypothetical protein	**1,26**	0,14	**1,12**	-0,05
SMc01510 (*hmuV*)	Putative hemin transport system ATP-binding ABC transporter	-0,06	**1,48**	**1,53**	-0,12
SMc01512 (*hmuT*)	Putative hemin binding periplasmic transmembrane protein	-0,34	**1,49**	**1,39**	0,09
SMc01513 (*hmuS*)^c^	Putative hemin transport protein	-0,93	**1,34**	**2,60**	-0,07
SMc01514	Conserved hypothetical protein	**-1,14**	**1,50**	**2,37**	-0,09
SMc01658 (*fhuF*)	Siderophore reductase	-0,39	**1,28**	**2,03**	-0,06
SMc01659 (*fhuP*)	Periplasmic component of ferrichrome and ferrioxamine B ABC transporter	-0,41	**1,85**	**2,60**	0,12
SMc01747 (*hmuP*)	Hypothetical protein, hemin uptake protein	-0,78	**1,29**	**2,23**	0,04
SMc01917 (*nuoE1*)	NADH dehydrogenase I chain E	0,03	**1,21**	**-1,13**	-0,14
SMc02084 (*exbD*)	Probable biopolymer transport transmembrane protein	-0,68	**1,48**	**1,16**	-0,03
SMc02085 (*exbB*)^c^	Probable biopolymer transport transmembrane protein	-0,54	**1,82**	**1,55**	-0,01
SMc02726 (*shmR*)	Hemin-binding outer membrane receptor	-0,11	**2,02**	**2,84**	0,30
SMc03167	MFS-type transport protein	**1,09**	0,62	**1,27**	0,38

^a ^The subset S36 comprises 36 genes showing differential expression in the two transcriptome comparisons aimed to identify swarming responsive genes (i.e. semisolid vs. solid and semisolid vs. broth); ^b ^log_2 _(experiment signal/control signal). Values in bold face indicate that they meet both M and p criteria; ^c ^Genes validated by RT-qPCR (see Fig. 5B). SS, growth in semisolid MM (0.6% agar); S, growth in solid MM (1.3% agar); L, growth in liquid MM.

### Validation of the results from the microarray experiments by RT-qPCR

**Figure 5:**
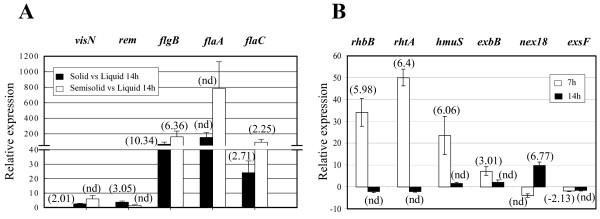
**Confirmation of the differential expression of selected genes in a *fadD *mutant of *S. meliloti *in response to different growth conditions as determined by quantitative real-time PCR**. A) The relative expression of surface responsive genes was calculated as the fold change between growth on solid MM (black bars) or semisolid MM (white bars) compared to growth in liquid MM after 14 hours of incubation. B) The relative expression of genes responding specifically to swarming inducing conditions was calculated as the fold change between growth on semisolid MM compared to growth on hard agar MM at 7 hours (white bars) or at 14 hours (black bars). For comparisons, fold changes in gene expression obtained in the microarray experiments (calculated as 2^M^) are shown in parenthesis. Results are averages from at least two independent biological experiments with three technical replicates. Error bars indicate standard error at 95% confidence. nd, not detected in the microarray experiments

To validate our microarray data we performed reverse transcription-quantitative PCR on several selected surface responsive genes as well as on genes showing response to swarming-specific conditions. Among surface responsive genes, we analyzed the expression of several motility genes belonging to different classes of the *S. meliloti *flagellar regulon [28,29] on solid, semisolid and in liquid MM after 14 h of growth: the *visN *and *rem *genes coding for master regulators of class IA and class IB, respectively; the *flgB *gene as a representative of class II genes, encoding a flagellar basal-body rod protein; and the class III genes *flaA *and *flaC*, encoding the principal and secondary flagellins, respectively. In our transcriptomic study *flaA *was not present in the list of differentially expressed genes. Since many motility genes were up-regulated in response to surface growth and FlaA is the main component of the flagellum, we hypothesized that the fact that this gene does not show differential expression in our study, could be due to the limitations inherent to the microarray approaches. Therefore, we decided to include *flaA *in the RT-qPCR studies. All five of the motility genes analyzed showed surface specific induction (Fig. 5A), thereby confirming the microarray results. Interestingly, the RT-qPCR analyses revealed that, except for *rem*, the motility genes analyzed showed higher induction values on semisolid than on solid media, which is in agreement with the existence of a higher motility activity under swarming inducing conditions.

To confirm the differential expression of genes showing response to swarming-specific conditions, we selected: four genes related to iron uptake and metabolism (*rhbB, rhtA, hmuS *and *exbB*) which showed early induction (7 h) in semisolid vs. solid; *nex18*, a symbiotically induced gene showing late (14 h) up-regulation in semisolid vs. solid; and *exsF*, a gene coding for a putative two-component response regulator with sequence similarity to CheY, and found as an early down-regulated gene in swarm cells compared to cells grown on solid MM. The expression of these six genes was determined on solid and semisolid MM after 7 and 14 hours of growth. As shown in Fig. 5B, once more, the RT-qPCR results confirmed the microarrays data.

As detailed above, we have macroscopic evidence that under our experimental conditions (spread plating on semisolid MM) cells of 1021FDC5 show swarming (Fig. 3). However, to test whether the genes differentially expressed under these conditions could truly be considered swarming-specific, we analyzed and compared the expression of *rhbB*, *rhtA *and *hmuS *by RT-qPCR from cells present in the border of swarming colonies obtained in standard swarming assays and cells from a colony grown on solid MM. The results confirmed the up-regulation of these genes in swarming cells vs non-swarming cells with relative expression values of 5.72 ± 0.54 for *rhbB*, 4.61 ± 0.38 for *rhtA *and 4.41 ± 0.69 for *hmuS*. The differences in the induction values found for these genes between cells spread plated on semisolid MM (Fig. 5B) and cells from the border of a typical swarming colony could be explained by differences in the growth phase of the two samples. Nevertheless, these data indicate that our experimental approach is adequate for the identification of swarming-specific genes.

### Role of pSymA, rhizobactin-related genes and iron in *S. meliloti *swarming

**Figure 6:**
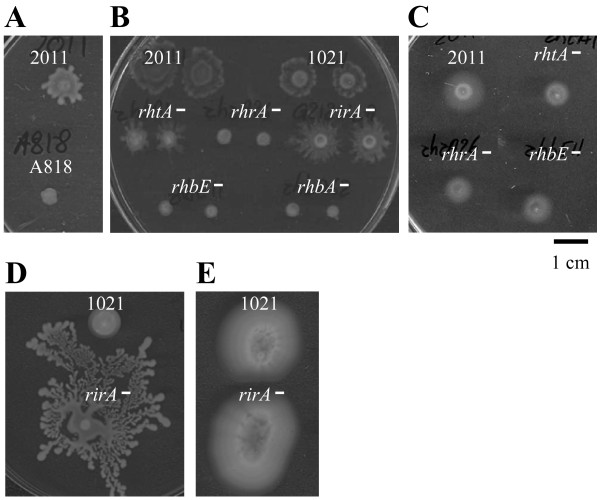
**Effect of pSymA, rhizobactin-related genes, and iron concentration on the motility of *S. meliloti***. A) Swarming test of wild type Rm2011 and a pSymA-cured derivative strain (SmA818). B) Swarming assay of mutants in the biosynthesis and transport of rhizobactin 1021 and in the rhizobial iron regulator *rirA*. C) Swimming test of Rm2011 and rhizobactin 1021-related mutants in Bromfield (0.3% agar). D) Swarming and E) swimming tests of Rm1021 and the *rirA *mutant in MM containing high iron concentration (220 μM). Photographs were taken either at 48 hours (A, B, and C) or 5 days (D and E) after inoculation and are representative of three replicates from at least three different experiments.

Since the proportion of genes belonging to pSymA present in the subset S36 of swarming-responsive genes was higher than expected, we investigated whether this megaplasmid played any role in surface translocation. The swarming ability of SmA818, a *S. meliloti *strain cured of pSymA, was tested. In contrast to the parental strain Rm2011, SmA818 did not show swarming in any of the numerous assays performed (Fig. 6A). Mutagenesis-based approaches have revealed that a wide variety of genes are involved in swarming [7,8,19]. Since pSymA harbours more than one-fifth of the genes present in the *S. meliloti *genome, the finding that loss of this megaplasmid results in loss of swarming, might be not surprising. However, this result prompted us to investigate which genes of pSymA could play a role in triggering conditional swarming in *S. meliloti*.

Among the pSymA swarming-specific induced genes were those involved in the biosynthesis and transport of the siderophore rhizobactin 1021 [30]. In *E. coli*, mutations in most of the genes involved in the utilization of the siderophore enterobactin strongly inhibit swarming [7]. Likewise, in *P. putida*, mutants either in the siderophore pyoverdine or in the FpvA siderophore receptor have been shown to be defective in surface motility [44]. Hence, the swarming-defective phenotype observed in SmA818 could be due to the lack of rhizobactin-related genes. To test this, swarming assays were performed with mutants affected in either of the two different rhizobactin 1021 biosynthesis genes (*rhbA *and *rhbE*), a mutant lacking the RhtA outer membrane receptor for the siderophore, and with a *rhrA *mutant strain lacking the AraC-like regulator which positively regulates the production and transport of rhizobactin 1021. Additionally, we also looked at the swarming phenotype of a *rirA *mutant. RirA has been demonstrated to be the general regulator of the iron response in *S. meliloti*, including genes involved in the biosynthesis and transport of rhizobactin 1021 [42,43]. In our microarrays, *rirA *appeared to be induced 2-fold in growth on semisolid vs. solid media after 14 hours of incubation (see additional file 2). As shown in Fig. 6B, neither the mutants in the rhizobactin biosynthesis genes (*rhb*) nor the *rhrA *mutant were able to swarm, while the absence of either the RhtA siderophore receptor or the RirA regulator did not prevent swarming. The motility defect shown by the *rhb *and *rhrA *mutants was specific for swarming since assays performed in Bromfield and MM (0.3% agar) showed that these strains were able to swim (Fig. 6C). Thus, the motility phenotypes shown by the *rhb *and *rhrA *mutants suggest that either rhizobactin-mediated iron uptake or rhizobactin *per se *play a role during swarming in *S. meliloti *Rm2011. In *P. putida*, the defect in swarming shown by mutants unable to synthesize the siderophore pyoverdine could be restored by adding different sources of iron, suggesting that the intracellular iron level rather than the siderophore is the functional signal for swarming in this bacterium [44]. To test whether the lack of surface motility in the *rhb *and *rhrA *mutants could be due to iron deficiency, increasing concentrations (22, 220, and 2200 μM) of either FeCl_3 _or the iron chelate ferric citrate, whose uptake is independent on siderophore, were added to the media. None of these conditions could restore surface translocation in the mutants (data not shown), with the highest concentration used being inhibitory of cell growth. This result indicated that low intracellular iron levels were not responsible for the swarming deficiency of the *rhb *and *rhrA *mutants, and that the presence of rhizobactin 1021 is important for triggering swarming in *S. meliloti*. Furthermore, the fact that the *rhtA *mutant which is defective in rhizobactin 1021 utilization [30], still swarms (Fig. 6B) suggests that the function played by rhizobactin 1021 in swarming is exerted outside the cell. Rhizobactin 1021 is a citrate-based dihydroxamate siderophore structurally similar to schizokinen with the only but important difference that rhizobactin 1021 contains a long-chain fatty acid ((E)-2-decenoic acid) that gives the siderophore an asymmetric structure and amphiphilic properties [45]. The role of the decenoic acid residue in rhizobactin 1021 function has not been studied, although it has been proposed to be important during the membrane translocation of the ferric complex by making the molecule more mobile. Considering our results, it is tempting to speculate that the surfactant properties of rhizobactin 1021 may promote surface translocation in *S. meliloti*. Similarly, the biosurfactant activity associated to long-chain AHLs produced by *R. etli *has been proved to play a direct role in surface movement of swarmer cells, adding a new function to these well known signalling molecules [18]. Curiously and in support of our hypothesis, *S. meliloti *GR4 which is not able to swarm on semisolid MM, does not produce siderophores in liquid MM as determined by the CAS assay (data not shown).

The restoration of surface motility of the rhizobactin-defective mutants was attempted by adding concentrated supernatants containing rhizobactin 1021. Although the addition of these supernatants functioned in iron nutrition bioassays restoring the growth of *rhb *mutants, they failed to promote swarming of the mutants and even hampered this surface motility of the wild type and *fadD *mutant strains (data not shown). This result might be due to the negative effect on swarming of supraoptimal concentrations of nutrients or compounds excreted by Rm2011.

To further confirm that the presence of rhizobactin 1021 is important for triggering swarming in *S. meliloti*, the motility phenotypes of Rm1021 and the *rirA *mutant were tested under iron-replete conditions as it has been reported that these conditions inhibit rhizobactin 1021 production in the wild type but not in the mutant [43]. CAS assays were performed to determine siderophore concentrations in the supernatants of these two strains under different growth conditions. We found that the wild type and the *rirA *mutant produced similar amounts of siderophore when cells were cultivated in MM containing 22 μM of FeCl_3 _(data not shown). The presence of 220 μM of FeCl_3 _abolished siderophore production in Rm1021 but not in the *rirA *mutant (data not shown). Hence, swimming and swarming assays were performed in MM containing 220 μM of FeCl_3_. No differences in swimming were observed between the two strains (Fig. 6E). However, swarming by Rm1021 was inhibited at this iron concentration but not that by the *rirA *mutant in which swarming seemed even to be enhanced compared to lower iron concentrations (Fig. 6D). This result not only supports that in *S. meliloti *Rm1021 rhizobactin 1021 is required for swarming but also suggests that iron and *rirA *play a role in the control of this multicellular phenotype. The concentration of iron in the medium has been shown to be decisive for swarming in several bacteria [44,46,47]. In *S. meliloti *strain Rm1021, like in *Pseudomonas *spp., an excess of iron inhibits swarming, an effect that in *S. meliloti *could be due at least in part to the inhibition of rhizobactin 1021 production. On the other hand, the enhanced motility shown by the *rirA *mutant under high iron conditions suggests that additional genes controlled by this regulator might be involved.

### The lack of a functional *fadD *gene restores swarming in pSymA-cured and rhizobactin-defective strains, and allows swarming under high-iron conditions

**Figure 7:**
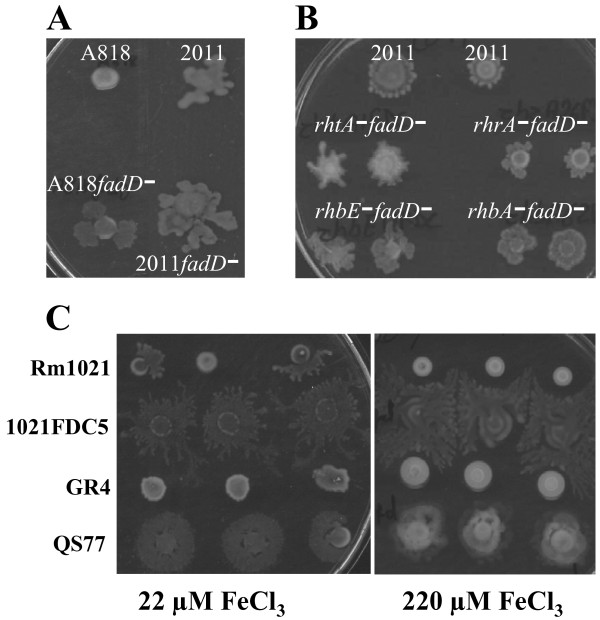
**Role of pSymA, rhizobactin-related genes, and iron concentration on *fadD*-dependent swarming of *S. meliloti***. Swarming tests of *fadD*-derivative mutants of Rm2011 and the pSymA-cured strain A818 (A), of double mutants lacking *fadD *and rhizobactin 1021-related genes (B), and wild type and *fadD*-derivative mutants under standard (22 μM FeCl_3_) and high iron conditions (220 μM FeCl_3_) (C). Photographs were taken 48 hours after inoculation and are representative of three replicates from at least three different experiments.

As described above, pSymA and at least the rhizobactin 1021-related genes *rhb *and *rhrA *are required for swarming in *S. meliloti *Rm1021. To investigate if these genes are also important in the surface motility shown by the *fadD *mutant, swarming assays were performed with the pSymA-cured strain SmA818 in which the *fadD *was inactivated as well as with double mutants *rhbfadD *and *rhrAfadD*. As shown in Fig 7A and 7B, the lack of a functional *fadD *gene restored surface motility in all the swarming-deficient strains. Thus, although rhizobactin biosynthesis and regulation genes were found to be up-regulated in the *fadD *mutant under swarming inducing conditions, these genes are not required for this surface motility in this genetic background. A possible explanation for these findings could be that the signal transduction pathway leading to the induction of the rhizobactin genes is not altered in the *fadD *mutant. A recent microarray analysis performed in our group supports this hypothesis. The comparison of the transcriptome of the wild type strain with that of the *fadD *mutant under swarming-inducing conditions after 7 and 14 hours of growth, revealed only 11 differentially expressed genes (including the up-regulation of the *npt *gene in the *fadD *mutant) (data not shown). Neither *rhb *genes nor *rhrA *were amongst them, suggesting that these genes show similar expression levels under swarming inducing conditions in both genetic backgrounds. The finding that rhizobactin-related genes are not essential for swarming in the *fadD *mutant could be explained if the function played by iron/rhizobactin 1021 in the control of swarming in Rm1021 (as a surfactant or signal molecule) could be exerted in the *fadD *mutant by a different and unknown compound which is not present or inactive in the wild-type strain.

We also tested if the presence of high iron concentrations prevents swarming in a *fadD *mutant as it does in 1021. Swarming assays were performed on semisolid MM containing 220 μM of FeCl_3 _with 1021 and GR4 as wild type strains, and their corresponding *fadD*-derivative mutants. As shown in Fig. 7C, swarming was never observed in GR4 but always in the *fadD *mutant QS77. As already mentioned, in 1021 swarming was observed at a certain frequency on MM containing 22 μM of FeCl_3 _and never observed under high iron conditions but its corresponding *fadD *mutant showed swarming at both iron concentrations similar to that found for the *rirA *mutant. However, in contrast to the *rirA *mutant, the iron-independent swarming phenotype shown by the *fadD *mutant cannot be explained by differences in the production of rhizobactin 1021 since the *fadD *mutant, like the wild type, inhibits siderophore production under high iron conditions (data not shown). Therefore in *S. meliloti*, the lack of a functional *fadD *gene relieves the control that iron has over swarming as well as the dependence on rhizobactin 1021 for this surface motility. A possibility worth investigating is if fatty acid derivatives, whose concentration is dependent on FadD activity but not iron-responsive, could replace siderophore function during swarming. Likewise, future investigations should address a possible connection between the *rirA *and *fadD *regulatory networks that could explain the iron-insensitivity of swarming shown by the *fadD *and *rirA *mutants.

## Conclusions

To the best of our knowledge, the present work represents the first global gene expression analysis of rhizobium growth on surfaces, including swarming inducing conditions. The results reveal that the physiology of *S. meliloti *cells growing on the surface of agar media is significantly different from that of cells growing in broth, with the differential expression of more than a thousand genes. It is tempting to speculate that these major changes in gene expression could also take place in rhizobium during colonization of root surfaces, an important prerequisite for nodule formation. Thus, the approach used in this study may be helpful to identify genes and regulatory mechanisms that could be crucial during the early stages of the rhizobium-legume symbiosis and it could serve as a model for studying gene expression in different plant-associated bacteria.

The surface motility shown by several *expR*-deficient strains in this work indicates that the role played by this LuxR-type regulator in swarming by *S. meliloti *needs to be re-examined. Moreover, the genomic analysis under swarming-inducing conditions allowed the identification of environmental signals (surface contact and iron concentration) and genes that play important roles in the control of this surface motility in a wild type strain of *S. meliloti*. Furthermore, the results suggest that rhizobactin 1021 plays a role in swarming although the requirement for rhizobactin-related genes and the inhibition of this surface motility by an excess of iron can be circumvented in a *fadD *mutant. Future work should focus on investigating the specific role of rhizobactin 1021 in swarming of *S. meliloti *as well as to identify why the lack of a functional *fadD *gene allows surface translocation of bacterial cells under conditions which negatively influence this type of multicellular migration.

## Methods

### Bacterial strains and growth conditions

Strains used in this study are listed in Table 3. *E. coli *strains were grown in Luria-Bertani (LB) medium [48] at 37°C; *S. meliloti *strains were grown at 30°C in TY complex medium [49] or in minimal medium (MM) containing glutamate (6.5 mM), mannitol (55 mM), mineral salts (K_2_HPO_4_, 1.3 mM; KH_2_PO_4_. 3H_2_O, 2.2 mM; MgSO_4 _7H_2_O, 0.6 mM; CaCl_2 _2H_2_O, 0.34 mM; FeCl_3 _6H_2_O, 0.022 mM; NaCl, 0.86 mM) and vitamins (biotin (0.2 mg/L); calcium pantothenate (0.1 mg/L)) [50]. Standard MM contains 22 μM FeCl_3. _When a different concentration or source of iron was required, 100-fold concentrated stock solutions of either FeCl_3 _or ferric citrate were prepared and added to MM without iron. To test the ability to use oleate as sole carbon source, MM was used in which glutamate and mannitol were replaced with 2 mM NH_4_Cl and 5 mM oleate, respectively. When required, antibiotics were added at the following final concentrations: for *E. coli*, streptomycin (Sm) 50 μg/ml, spectinomycin (Sp) 100 μg/ml, kanamycin (Km) 50 μg/ml, and ampicillin (Ap) 200 μg/ml; for *S. meliloti*, Sm 200 μg/ml, Km 200 μg/ml, rifampin (Rif) 100 μg/ml, and neomycin sulphate (Nm) 100 μg/ml. To improve reproducibility, all liquid cultures of *S. meliloti *were routinely initiated from glycerol stocks.

**Table 3:** Bacterial strains and plasmids used

Strain or plasmid	Relevant characteristics^a^	Reference or source
*Escherichia coli*		
DH5α	*supE*44, Δ*lacU*169, *f*80, *lacZ*ΔM, *recA*1, *endA*1, *gyrA*96, *thi*1, *relA*1, *5hsdR*171	Bethesda Research Lab^®^
S17.1	*thi*, *pro*, *recA*, *hsdR*, *hsdM*, Rp4Tc::Mu, Km::Tn*7*; Tp^r^, Sm^r^, Sp^r^	[57]
*Sinorhizobium meliloti*		
GR4	Wild type	[58]
QS77	GR4 (*fadD*::Tn*5*), Km^r^	[16]
Rm1021	SU47 *expR102*::IS*Rm*2011-1, Sm^r^	[59]
1021FDC5	Rm1021 (Δ*fadD*::Km), Sm^r ^Km^r^	This work
1021FDCSS	Rm1021 (Δ*fadD*::SmSp), Sm^r ^Sp^r^	This work
Rm2011	SU47*expR102*::IS*Rm*2011-1, Sm^r^	[60]
2011FDC	Rm2011 (Δ*fadD*::SmSp), Sm^r ^Sp^r^	This work
SmA818	Rm2011 pSymA cured, Sm^r^	[61]
A818FDC	SmA818 (Δ*fadD*::SmSp), Sm^r ^Sp^r^	This work
2011rhbA62	Rm2011 (*rhbA::*Tn*5lac*), Sm^r ^Rif^r ^Nm^r^	[30]
2011rhbAFDC	2011rhbA62 (Δ*fadD*::SmSp), Sm^r ^Sp^r ^Rif^r ^Nm^r^	This work
2011rhbE11	Rm2011 (*rhbE::*Tn*5lac*), Sm^r ^Rif^r ^Nm^r^	[30]
2011rhbEFDC	2011rhbE11 (Δ*fadD*::SmSp), Sm^r ^Sp^r ^Rif^r ^Nm^r^	This work
2011rhrA26	Rm2011 (*rhrA::*Tn*5lac*), Sm^r ^Rif^r ^Nm^r^	[30]
2011rhrAFDC	2011rhrA26 (Δ*fadD*::SmSp), Sm^r ^Sp^r ^Rif^r ^Nm^r^	This work
2011rhtA1	Rm2011 (*rhtA::*Tn*5*), Sm^r ^Rif^r ^Nm^r^	[30]
2011rhtAFDC	2011rhtA1 (Δ*fadD*::SmSp), Sm^r ^Sp^r ^Rif^r ^Nm^r^	This work
G212rirA	Rm1021 (lac^-^, *rirA*::Km), Sm^r ^Km^r^	O'Connell, M.
G212rirAFDC	G212rirA (Δ*fadD*::SmSp), Sm^r ^Km^r^	This work
Plasmids		
pBSKS(+)	Cloning vector; Ap^r^	Stratagene
pHP45Ω	Plasmid containing Sm/Sp cassette; Ap^r^, Sm^r^, Sp^r^	[62]
pHP45Ω Km	Plasmid containing Km cassette; Ap^r^, Km^r^	[63]
pK18*mobsacB*	Suicide plasmid; Km^r^	[51]
pBBRD4	pBBR1 MCS-3 derivative containing the *fadD *gene of *S. meliloti *GR4; Tc^r^	[16]
pBSDIL12	pBSKS derivative containing the *fadD *gene of *S. meliloti *GR4; Ap^r^	This work
pBS12.6Km	pBSDIL12 in which the *fadD *gene has been deleted and interrupted with a Km cassette; Ap^r ^Km^r^	This work
pK18fadDCKm	pK18*mobsacB *carrying the fadD mutated version of pBS12.6Km;	This work
pK18fadDCSS	pK18fadDCKm in which the Km cassette interrupting the *fadD *gene has been substituted by a Sm/Sp cassette	This work

### Construction of *S. meliloti fadD *mutants

The *fadD*^- ^strain 1021FDC5 used in the microarray experiments was obtained by allelic exchange. A disrupted version of the *fadD *gene was constructed by deleting an internal fragment and inserting a kanamycin resistance cassette. Firstly, a *Kpn*I/*Xba*I fragment harbouring the *fadD *gene of *S. meliloti *was subcloned from pBBRD4 [16] into pBluescript to give pBSDIL12. After removal of a *Bam*HI site from the polylinker of pBSDIL12, an internal *Bam*HI fragment of 300 bp of the *fadD *gene was replaced with a 2.2 kb *Bam*HI fragment containing the Km^R ^cassette from pHP45Ω-Km to give pBS12.6Km. This construction was digested with *Kpn*I, treated with T4 DNA polymerase (Roche Biochemicals) to make blunt ends, and then digested with *Xba*I to isolate the Km^R ^fragment which was then cloned into the suicide vector pK18*mobsacB *previously digested with *Sma*I/*Xba*I, to give pK18fadDCKm. This plasmid was introduced by conjugation into *S. meliloti *1021 and allele replacement events were selected as described previously [51].

The *fadD*^- ^strain 1021FDCSS was obtained following the same procedure as for 1021FDC5 with the only difference that the Km^R ^cassette present in pK18fadDCKm was substituted by the Sm^R^/Sp^R ^cassette from pHP45Ω to give pK18fadDCSS. The *fadD *mutation present in 1021FDCSS was transferred into different strain backgrounds by generalized transduction of 1021FDCSS using phage ôM12 as described previously [52]. All the different *fadD *mutants obtained were confirmed by Southern hybridization with a specific probe.

### Swarming and swimming assays

Swarming assays were carried out as described in Soto *et al*. [16]. Briefly, *S. meliloti *cells grown in TY broth to late logarithmic phase (optical density (OD) at 600 nm = 1-1.2) were pelleted, washed twice in MM and resuspended in 0.1 volume of the latter medium. 2 μl aliquots of this bacterial suspension (ca. 2 × 10^7^cells) were dispensed onto the surface of swarm plates and allowed to dry for 10 min. Swarm plates were prepared with 20 ml of MM containing 0.6% purified agar (Pronadisa), and air dried at room temperature for 15 min. Incubation periods of 14 to 20 h at 30°C, were enough to observe swarming. To complement swarming in rhizobactin-defective mutants, a concentrated supernatant containing rhizobactin 1021 was prepared as described by Lynch *et al*. [30] from wild-type strain Rm2011 grown to stationary phase in either TY broth with 200 μM 2, 2'-dipyridyl or MM broth with 2 μM 2, 2'-dipyridyl. Before its use in swarming assays, the presence of siderophore in the supernatants was checked in iron nutrition bioassays as described by Lynch *et al*. [30]. Two complementation approaches were used: 1) a well was cut in the center of a swarm plate and 100 μl of the rhizobactin containing supernatant was added. Aliquots of the wild type strain and rhizobactin-defective mutants prepared as described above were placed onto the surface of the swarm plate surrounding the well; 2) cells of the wild type strain and rhizobactin-defective mutants were grown in TY broth, pelleted, washed twice in MM and resuspended in 0.1 volume of the rhizobactin containing supernatant. Finally, 2 μl aliquots of this bacterial suspension were assayed for surface motility on swarm plates.

Swimming plates were prepared with either Bromfield medium (0.04% tryptone, 0.01% yeast extract, and 0.01% CaCl_2_.2H_2_O) containing 0.3% Bacto agar or with MM containing 0.3% purified agar. Plates were inoculated with 3 μl droplets of rhizobial cultures grown in TY, and incubated at 30°C for 2 to 5 days.

### Determination of bacterial growth curves

Bacterial growth curves of *S. meliloti *1021FDC5 were determined in liquid, semisolid (0.6% purified agar) and solid (1.3% purified agar) MM. A preinoculum was grown in 20 ml of TY broth to late logarithmic phase (OD_600 nm _= 1-1.2). After incubation, cells were pelleted, washed twice in MM and resuspended in 2 ml of the latter medium. For growth curves in liquid MM, Erlenmeyer flasks (250 ml) containing 50 ml of liquid MM were inoculated with 0.5 ml of the rhizobial suspension (approximately 10^8 ^cells/ml) and incubated at 30°C with continuous shaking (190 r.p.m.). For growth curves in plates, aliquots of 0.1 ml of the rhizobial suspension were used to sow MM plates (approximately 10^9 ^cells/plate). This size of inoculum was used to ensure that on semisolid and solid MM plates, the same density of cells per surface area was applied as in standard swarming assays (10^7 ^cells per 0.2 cm^2^). The rhizobial suspension was evenly spread over the surface of semisolid and solid MM plates, allowed to dry for 10 min and then inverted and incubated at 30°C. This sampling on plates was preferred over inoculation with droplets, to minimize heterogeneity among cells. Samples from liquid cultures and plates were collected at different time points for cell count determination. Cells grown on plates were harvested by scraping the surface with 2 ml of sterile liquid MM.

### RNA isolation and synthesis of labelled cDNA

For RNA isolation, cells from 18 ml of broth culture or grown on the surface of 3 plates were harvested, washed with sarkosyl 0.1% and cell pellets were immediately frozen in liquid nitrogen and conserved at -80°C until RNA isolation. For microarray hybridization and reverse transcription quantitative real-time PCR (RT-qPCR), RNA was isolated using the Qiagen RNeasy RNA purification kit (Qiagen) following the manufacturer's instructions. Residual DNA was removed with RNase-free Dnase I Set (ROCHE). The quality of the RNA was checked on 1.4% agarose gel electrophoresis.

Cy3- and Cy5-labelled cDNAs were prepared according to DeRisi *et al*. [53] from 15 μg of total RNA. Three slide hybridizations were performed using the labelled cDNA synthesized from each of the RNA preparations from three independent bacterial cultures.

### Microarray hybridization, image acquisition and data analysis

Sm6koligo microarrays were purchased from A. Becker (University of Bielefeld, Bielefeld, Germany). Hybridizations were performed as described previously [21,37]. For image acquisition a GenePix 4100A Scanner (Axon Instruments, Inc., Foster City, CA, USA) was used. Quantifications of mean signal intensities for each spot were determined using the GenePix Pro 5.0 software (Axon Instruments, Inc.). Normalization and t-statistics were carried out using the EMMA 2.6 microarray data analysis software developed at the Bioinformatics Resource Facility Center for Biotechnology, Bielefeld University http://www.genetik.uni-bielefeld.de/EMMA/[54]. Three independent biological replicates were performed for each experiment. Genes were regarded as differentially expressed if they showed p ≤ 0.05, A ≥ 7 and M ≥ 1 or M ≤ -1 (A, average signal to noise; M value is log_2 _experiment/control ratio) in any of the experiments performed. Detailed protocols and raw data resulting from the microarray experiments have been deposited in the ArrayExpress database with the accession number E-MEXP-1953.

### Reverse transcription quantitative real-time PCR (RT-qPCR)

Total RNA (1 μg) treated with RNase-free Dnase I Set (ROCHE) was reversely transcribed using Superscript II reverse transcriptase (INVITROGEN) and random hexamers (ROCHE) as primers. Quantitative real-time PCR was performed on an iCycler iQ5 (Bio-Rad, Hercules, CA, USA). Each 25 μl reaction contained either 1 μl of the cDNA or a dilution (1:10.000, for amplification of the 16S rRNA gene), 200 nM of each primer and iQ SyBrGreen Supermix (BioRad). Control PCR reactions of the RNA samples not treated with reverse transcriptase were also performed to confirm the absence of contaminating genomic DNA. Samples were initially denatured by heating at 95°C for 3 minutes followed by a 35-cycle amplification and quantification program (95°C for 30 s, 55°C for 45 s, and 72°C for 45 s). A melting curve was conducted to ensure amplification of a single product. The oligonucleotide sequences for qPCR are listed in additional file 3. The efficiency for each primer pair (E) was determined by running 10-fold serial dilutions (4 dilution series) of Rm1021 genomic DNA as template and generating a standard curve by plotting the log of the dilution factor against the C_T _value during amplification of each dilution. Amplification efficiency is calculated using the formula (E = [10^(1/*a*)^-1] × 100) where *a *is the slope of the standard curve.

The relative expression of each gene was normalized to that of 16 S rRNA and the analysis of results was done using the comparative critical threshold (ΔΔC_T_) method [55].

### CAS siderophore assay

The determination of siderophores in liquid cultures was performed using the Chrome azurol S (CAS) assay solution described by Schwyn and Neilands [56]. Supernatants of *S. meliloti *cultures grown in MM containing different concentrations of FeCl_3 _were mixed 1:1 with the CAS assay solution. After reaching equilibrium, the absorbance was measured at 630 nm.

## Abbreviations

AHL: N-acyl-homoserine lactones; TTSS: type III secretion system; MM: minimal medium; EPS: exopolysaccharide; RT-qPCR: reverse transcription-quantitative polymerase chain reaction; (Sm): Streptomycin; (Sp): Spectinomycin; (Km): Kanamycin; (Ap): Ampicillin; (Rif): Rifampin; (Nm): Neomycin sulphate; OD: optical density; CAS: Chrome azurol S.

## Authors' contributions

JN and AD-F performed experiments, analyzed data and participated in the writing of the manuscript. PvD performed experiments and contributed to the writing. CVA-G and VC performed experimental work. JS and JO helped to coordinate the study and contributed to the writing. MJS designed research, analyzed data and wrote the manuscript. All authors have read and approved the final manuscript.

## Supplementary Material

Additional file 1 **Genes differentially expressed in response to surface growth and/or swarming-specific conditions in *S. meliloti *1021FDC5**. Tabular data (.xls) list the 1166 genes showing differential expression in any of the six comparisons performed in this study. Only M values above 1 or below -1 with p ≤ 0.05 are shown. The category in the Venn diagram (A-G in Fig. 4) to which each gene belongs is also indicated. **L**, growth in **l**iquid MM; **S**, growth on **s**olid MM (1.3% agar); **SS**, growth on **s**emi **s**olid MM (0.6% agar). Time of incubation is shown in parenthesis. Click here for file

Additional file 2 **Swarming-responsive genes identified in *S. meliloti *1021FDC5**. Tabular data (.xls) list the 294 genes showing differential expression in response to swarming-specific conditions. Only M values above 1 or below -1 with p ≤ 0.05 are shown. The category in the Venn diagram (A-G in Fig. 4) to which each gene belongs is also indicated. **L**, growth in **l**iquid MM; **S**, growth on **s**olid MM (1.3% agar); **SS**, growth on **s**emi **s**olid MM (0.6% agar). Time of incubation is shown in parenthesis. Click here for file

Additional file 3 **Sequences of the oligonucleotides used for quantitative real-time PCR**. Table of data. Click here for file
