# Supplementary material for: Transcriptome profiling of a Sinorhizobium meliloti fadD mutant reveals the role of rhizobactin 1021 biosynthesis and regulation genes in the control of swarming
Source: BMC Genomics. 2010 Mar 8;11:157. doi: 10.1186/1471-2164-11-157 (PMC2848241; doi:10.1186/1471-2164-11-157)
Supplement: Additional file 3 — Sequences of the oligonucleotides used for quantitative real-time PCR. Table of data. [file 1471-2164-11-157-S3.DOC]

## Table 4 - Sequences of the oligonucleotides used for quantitative real-time PCR.

| **Gene** | **Forward Primer (5’ to 3’)** | **Reverse Primer (5’ to 3’)** |
| --- | --- | --- |
| SMc03224 (*16S*) | TCTACGGAATAACGCAGG | GTGTCTCAGTCCCAATGT |
| SMc03015 (*visN*) | TCCTTGATGCTGCTCTTC | CTCGGTCAGTTCGCATTC |
| SMc03046 (*rem*) | CGAAAGCCACATCAGCAAGC | ATTCCAGTCGATGCAGTAGCC |
| SMc03027 (*flgB*) | GAAAGCGTGCTTCAGAAC | CTGACTTCGGTCACATGC |
| SMc03037 (*flaA*) | CGATTATGTCAAGGTCCA | GCAATGGTGATGTCGATC |
| SMc03040 (*flaC*) | CCGACGGCAGCGTTACG | ATCCGCATTCACCGCCTTG |
| SMc01513 (*hmuS*) | ACATCAAGCAAGGACACG | CACTTGTCGAAGAACTGC |
| SMc02085 (*exbB*) | CTTCCGCATGATCTTTCG | AGTTCCAGCGTCTTCACG |
| SMa2402 (*rhbB*) | TGAACATCAACGTCGCTG | GGAGTAGAGACTGCTTGC |
| SMa2414 (*rhtA*) | CATCATCACGAAGAAGGG | TCGCTGTTATAGGTGACC |
| SMa1077 (*nex18*) | GTCAATTCAAGACGCTGG | GGTTTCAGCAGGTTTTCG |
| SMb20934 (*exsF*) | ATCGCTACTCTCGATCTC | TCGTTCAGGCAAGTCAAG |
